# The Role of the Anabolic Properties of Plant- versus Animal-Based Protein Sources in Supporting Muscle Mass Maintenance: A Critical Review

**DOI:** 10.3390/nu11081825

**Published:** 2019-08-07

**Authors:** Insaf Berrazaga, Valérie Micard, Marine Gueugneau, Stéphane Walrand

**Affiliations:** 1 UNH, Unité de Nutrition Humaine, CRNH, Université Clermont Auvergne, INRA, Auvergne, 63000 Clermont-Ferrand, France; 2 IATE Agropolymer Engineering and Emerging Technologies, Univ. Montpellier, INRA, CIRAD, Montpellier SupAgro, 34060 Montpellier, France; 3 Service de Nutrition Clinique, Centre Hospitalier Universitaire (CHU) Gabriel Montpied, 63000 Clermont-Ferrand, France

**Keywords:** plant-based proteins, animal-based proteins, older people, skeletal muscle, muscle protein synthesis, critical review

## Abstract

Plant-sourced proteins offer environmental and health benefits, and research increasingly includes them in study formulas. However, plant-based proteins have less of an anabolic effect than animal proteins due to their lower digestibility, lower essential amino acid content (especially leucine), and deficiency in other essential amino acids, such as sulfur amino acids or lysine. Thus, plant amino acids are directed toward oxidation rather than used for muscle protein synthesis. In this review, we evaluate the ability of plant- versus animal-based proteins to help maintain skeletal muscle mass in healthy and especially older people and examine different nutritional strategies for improving the anabolic properties of plant-based proteins. Among these strategies, increasing protein intake has led to a positive acute postprandial muscle protein synthesis response and even positive long-term improvement in lean mass. Increasing the quality of protein intake by improving amino acid composition could also compensate for the lower anabolic potential of plant-based proteins. We evaluated and discussed four nutritional strategies for improving the amino acid composition of plant-based proteins: fortifying plant-based proteins with specific essential amino acids, selective breeding, blending several plant protein sources, and blending plant with animal-based protein sources. These nutritional approaches need to be profoundly examined in older individuals in order to optimize protein intake for this population who require a high-quality food protein intake to mitigate age-related muscle loss.

## 1. Introduction

It is important to preserve skeletal muscle mass to maintain or improve metabolic homeostasis and physical function. In this context, the rates of protein synthesis and degradation in skeletal muscle constantly adapt in order to maintain muscle mass. Muscle protein synthesis and muscle protein breakdown rates are highly influenced by physical activity and food intake [1,2]. In healthy adults, dietary intake is generally associated with an increase in the plasma concentrations of nutrients and hormones causing an increase in protein synthesis and a decrease in protein breakdown rates, in particular in skeletal muscle [3,4,5]. However, older people develop a resistance to the stimulation of muscle protein synthesis following meal intake. This ‘anabolic resistance’ makes their body protein compartments, notably skeletal muscle, unable to compensate for protein losses during the post-absorptive period. Net protein loss at the skeletal muscle level progressively causes a generalized reduction in skeletal muscle mass and function called ‘sarcopenia’ [6,7,8]. Sarcopenia is recognized as an actual disease by the World Health Organization (WHO) and listed in the international classification of diseases. Physical impairments associated with sarcopenia result in an increased risk of falls, loss of independence, and reduced quality of life [8].

**Figure 1:**
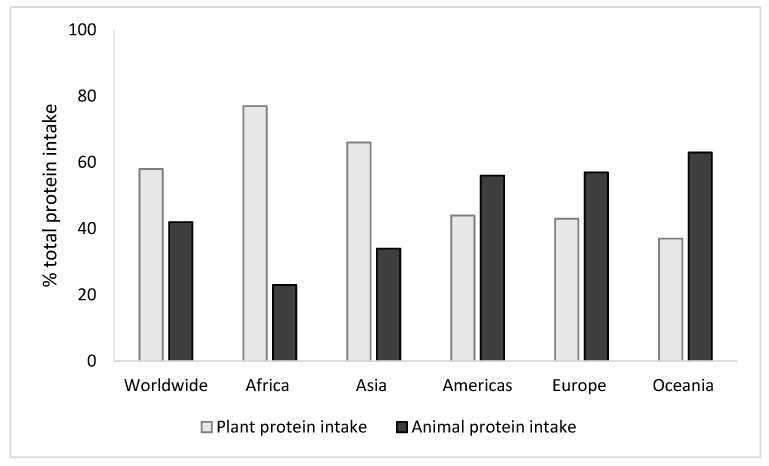
The percentage of dietary protein intake derived from plant and animal protein sources in different parts of the world [45].

Several studies have shown that the ability of muscle to respond to meal-associated anabolic stimuli, i.e., amino acids and insulin, is impaired during aging [9,10,11,12,13,14,15], which may explain the impaired anabolic response of muscles following food intake and especially protein intake. The literature logically highlights the importance of optimizing protein intake in the elderly, both by increasing protein quantity and improving protein quality, to overcome the reduced muscle anabolic response to food intake [7,16,17,18]. Several studies have evaluated the effect of consuming plant-based proteins on muscle protein metabolism in young, adult and old rats, pigs, and humans, compared to animal proteins, i.e., meat, milk, and its constitutive proteins (casein and whey proteins) [19,20,21,22,23,24,25,26,27,28,29,30,31,32,33,34,35,36,37,38,39,40,41,42,43,44]. A few of these studies have focused on the impact of plant-based foods [41], soy protein [42,43], or wheat protein [44] ingestion on protein synthesis at the whole body or skeletal muscle level in older individuals. The majority of these studies have reported that good-quality animal proteins have a greater ability to enhance muscle protein synthesis rate and support muscle mass than plant-based proteins [19,20,21,22,23,24,25,26,27,28,29,30,31,32,33,34,35,36,37,38,39,42,44]. However, worldwide, plant-based proteins contribute more to protein intake than animal-based proteins (Figure 1) [45]. Furthermore, older people generally eat less animal products, due to a blunted appetite for protein-rich foods, reduced chewing efficiency, metabolic abnormalities requiring a reduction of animal food products, and socio-economic factors. It is, therefore, necessary to develop plant-protein based foods adapted to older people’s needs. Plant-based diets are not just valuable for physical human health (including decreased risk of developing cancers, type 2 diabetes and cardiovascular diseases [46]) but are also more environmentally sustainable than animal-based diets, as recently reviewed by Lynch et al. [46].

The aim of this review is to evaluate the quality of plant- versus animal-based proteins and to assess the ability of plant-based proteins to stimulate muscle protein synthesis rates and help sustain skeletal muscle mass in healthy adults. Given the importance of maintaining skeletal muscle mass during aging, we focus on evaluating anabolic muscular response to intakes of plant-based proteins in older people, especially in physiopathological situations like sarcopenia.

## 2. Protein Quality of Plant- Versus Animal-Based Proteins

Dietary protein quality is assessed based on the essential amino acid composition of a protein as it relates to human needs and the ability of the protein to be digested, absorbed, and retained by the body [47]. The nutritional value of dietary proteins is, therefore, related to the bioavailability of its constitutive amino acids and depends on the efficiency of their metabolic utilization to meet the amino acid requirements necessary for growth and body protein turnover [48]. In 1989, the joint Food and Agriculture Organization of the United Nations/World Health Organization (FAO/WHO) Expert Consultation on Protein Quality Evaluation proposed measurable parameters based on the determination of nitrogen balance to evaluate the quality of dietary protein, i.e., protein digestibility, net protein utilization, biological value, chemical score, and digestibility corrected amino acid score [49].

Food protein quality as assessed by digestibility, net protein utilization, and biological value has so far been better for animal-based protein sources like meat, eggs, milk and its constituents than for plant-based protein sources like raw cereals and legumes (Table 1). The Protein Digestibility Corrected Amino Acid Score (PDCAAS) is a composite indicator of protein quality used to assess the ability of dietary protein to meet the body’s amino acid requirements [49]. This measure takes into account the essential amino acid composition of dietary protein as well as its true fecal digestibility. A given dietary protein cannot fully meet the body’s essential amino acid requirements when its PDCAAS is less than 100%. Apart for some soy protein isolates, the plant-based protein sources that have been tested to date are characterized by a PDCAAS that is below 100% and, therefore, lower than that of animal proteins. Wheat gluten is the plant-based protein with the lowest PDCAAS value at just 25% (Table 1). The lower PDCAAS of plant-based protein sources could be due to their lower digestibility compared to animal-based proteins [50] and/or to a deficiency in certain essential amino acids for body needs [51] (Table 2).

In addition to the PDCAAS, the FAO Expert Consultation on Dietary Protein Quality Evaluation in Human Nutrition suggested that proteins should be described based on their digestible amino acid content. Each amino acid should be considered as an individual nutrient, since digestibility may differ among amino acids [50]. Thus, in March 2013, the FAO proposed a protein quality score named the Digestible Indispensable Amino Acid Score (DIAAS), which takes into account the digestible amino acid content compared to a reference protein and its ileal digestibility. The FAO suggested using true ileal digestibility for each amino acid to calculate the DIAAS, rather than true fecal digestibility. Ileal digestibility better reflects the absorption of dietary amino acids since it does not take into account the nitrogen from the microbiota [50]. The plant-based proteins investigated so far are characterized by a DIAAS below 100%, which is, again, lower than that of animal proteins. The PDCAAS and DIAAS indexes give an indication of the first limiting amino acid of the protein. In legumes, such as soybeans, peas, faba beans and lentils, sulfur amino acids are the first limiting amino acids, whereas in cereals, such as wheat and maize, lysine is the first limiting amino acid (Table 2). The essential amino acids that do not get synthesized by the body are found at contents below the amino acid requirement recommended by the World Health Organization/Food and Agriculture Organization of the United Nations/United Nations University (WHO/FAO/UNU) for healthy adults. Consequently, the low essential amino acid content could limit protein synthesis [62]. This means that amino acid composition, protein digestibility, and availability are the determining factors for assessing dietary protein quality [63].

Plant-based proteins are less digestible than animal proteins [50]. This could be due to the different structure of plant versus animal proteins. The secondary structure of plant proteins is characterized by a high content in β-sheet conformation and a relatively low α-helix amount compared to that of animal proteins [64]. The high content in β-sheet conformation is likely related to its resistance to proteolysis in the gastrointestinal tract. Hence, the hydrophobic β-sheet structure of plant proteins that facilitates protein aggregation results in decreasing digestibility [64,65]. In addition, plant-based sources contain non-starch polysaccharides or fibers that impede the access of enzymes to proteins and could induce a decrease in protein digestibility [66]. The presence of some bioactive compounds named antinutritional factors, such as phytic acid, protease inhibitors, hemagglutinins, glucosinolates, tannins, and gossypol, could also affect the digestibility of plant-based protein sources [67]. For example, phytic acid, which is found in grains, seeds, and nuts (and is known to chelate minerals and thus reduce their bioavailability), can also interact with proteins, leading to decreased digestibility [68]. The enzymatic hydrolysis of phytic acid by phytase during food pretreatment or production (soaking, sprouting, germination, and use of endogeneous phytase) can lower its content in foods [69,70]. The protease inhibitors present in raw legumes, cereals, potatoes, and tomatoes disrupt protein digestion by complexing digestive enzymes, such as trypsin and chymotrypsin [67]. Heat treatment (e.g., drying, toasting and autoclaving) could significantly reduce trypsin inhibitory activity and therefore enhance the protein digestibility of treated plant-based products [59,67]. Heat-treated plant-based protein sources had 18% higher digestibility than unprocessed sources [58,59] (Table 1). In addition, the processing treatments used to commercially produce legume proteins, such as a legume protein concentrate or isolate, inactivate up to 80% of trypsin inhibitor activity in raw legume flour [67]. These treatments can improve digestibility up to a point that is comparable with animal proteins. The digestibility of pea protein concentrate was found to be 12% higher than that of untreated pea seeds and equivalent to that of casein (Table 1) [57,58].

In addition to the extent of protein digestion, a protein’s nutritional value also depends on its rate of digestion in the gastrointestinal tract [71]. Studies working on the kinetics of protein digestion and amino acid absorption have established the concept of ‘slow’ proteins (like casein) and ‘fast’ proteins (like whey) [71,72,73,74]. Boirie et al. [71] demonstrated that in healthy adult subjects who ingested milk protein fractions intrinsically labeled with L-[1-13C]leucine, whey proteins were digested more rapidly than native micellar casein. Whey proteins, which are highly soluble in acidic conditions, pass through the stomach and are rapidly hydrolyzed in the duodenum, causing rapid absorption and significant but transient aminoacidemia [71]. Nonetheless, caseins coagulate and are thus characterized by a slow and prolonged absorption of amino acids in the presence of gastric acidity [71]. Soy proteins are digested faster than casein and are slower than whey proteins [26,75]. Thus, the postprandial muscle protein synthesis rate did not increase to the same extent as whey proteins after the ingestion of soy proteins [42]. This could be due to the amino acid composition of soy proteins, chiefly their lower content of the protein anabolism regulator leucine, which stimulates protein synthesis and inhibits protein degradation [76,77]. Plant-based protein sources generally have a lower leucine content (7.1% ± 0.8%) than animal-based protein sources (8.8% ± 0.7% and even more than 10% in certain dairy proteins) [53,78]. Moreover, plant-based protein sources are deficient in certain essential amino acids for body needs (e.g., lysine in cereals [51]). When an essential amino acid is limiting, all other amino acids will not be properly used for protein synthesis and thus get deaminated and oxidized [62] and then irreversibly eliminated [79]. Limiting amino acids could, therefore, influence body protein accretion. Whole-body dietary protein retention has been assessed by measuring net postprandial protein utilization after the ingestion of 15N-labeled food proteins using a tracer technique [48,80,81]. The tracer technique is able to measure the whole body utilization of amino acids in terms of the entry of amino acids derived from labeled food proteins into the circulating metabolic amino acid pool and takes into account amino acid losses through the ileal and urinary routes [82,83]. Thus, studies have shown a higher deamination of amino acids derived from wheat protein (25% of ingested nitrogen deaminated) than milk protein (16%) within 8 h after intake by healthy subjects [48,80,81]. The net postprandial protein utilization value was, thus, lower for wheat proteins (66%) than for milk proteins (80%) [48,80,81]. Clinical and animal studies have also shown that amino acids from soy proteins were more degraded to urea than amino acids from casein or whey proteins and, consequently, were less available for protein synthesis in peripheral compartments, including skeletal muscle [26,33,42,84]. Nitrogen losses by deamination or intestinal loss and splanchnic nitrogen retention are higher after the ingestion of plant-based proteins than after the ingestion of animal-based proteins. As a result, the peripheral availability of amino acids derived from plant proteins is lower than that of animal proteins [85,86]. The metabolic fates of amino acids derived from plant and animal proteins are thus different, leading to metabolic differences in peripheral tissues like skeletal muscle.

## 3. Anabolic Properties of Plant-Based Proteins: Consequences on Muscle Protein Metabolism

There is a long history of studies evaluating acute muscle protein synthesis response or long-term changes in lean and skeletal muscle mass in response to the ingestion of plant-based protein sources. The bulk of these studies have investigated young-adult animal models or human subjects (Table 3 [23,24,26,27,28,29,30,31,33,34,35,37,38,39,40,87,88,89]), but a few have been carried out in situations corresponding to muscle loss, such as muscle loss in older subjects (Table 3 [42,43,44,88,89,90,91,92,93,94,95,96,97]).

### 3.1. Acute Clinical Studies on Plant- versus Animal-Based Proteins Enrolling Young and Older Subjects

Wilkinson et al. [40] and Tang et al. [75] evaluated the effect of an acute intake of plant- versus animal-based proteins on postprandial stimulation of muscle protein synthesis in young subjects. Wilkinson et al. [40] showed that in young men who performed resistance exercise, the consumption of skimmed milk was characterized by a 43% higher muscle protein synthesis rate than in subjects who in the same condition consumed an isonitrogenous and isocaloric drink containing soy protein isolate. Tang et al. [75] studied the postprandial muscle protein synthesis response to the ingestion of a beverage containing either whey hydrolysate, micellar casein, or soy protein isolate in resting conditions and after a resistance exercise in young men [75]. Note that in this study, all drinks result in an equivalent content of essential amino acids (10 g). In resting conditions, the muscle protein synthesis rate after the ingestion of a soy protein drink was 66% higher than that obtained after the ingestion of the beverage containing casein and 14% lower than that induced by the beverage containing whey proteins [75]. These differences might be related to protein digestion rates, which are faster for soy and whey proteins than for casein [26,71].

In older adults, the muscle protein synthesis rate was 30–40% lower following the consumption of a given quantity of soy or wheat protein hydrolysates than with whey protein isolate or micellar casein [42,44]. Yang et al. [42] showed that the consumption of soy protein isolate in elderly men in resting conditions did not stimulate the muscle protein synthesis rate, which remained lower than that induced by the consumption of the same amount of whey protein isolate. Note that this observation was reported regardless of the quantity of protein ingested (20 g or 40 g). These differences might be related to a lower postprandial leucinemia and higher amino acid oxidation following the consumption of soy proteins compared to whey proteins. Gorissen et al. [44] confirmed the lower anabolic properties of plant-based proteins compared to milk proteins. More specifically, the muscle protein synthesis rate was lowered in older men following the ingestion of 35 g of wheat protein hydrolysate than after the ingestion of 35 g of micellar casein.

All of these studies evaluated the effect of an acute intake of a bolus of plant-based proteins on the postprandial stimulation of muscle protein synthesis (Table 3) [40,42,44,75]. Several studies have also evaluated the effect of a chronic intake of plant-based proteins on changes in lean or skeletal muscle mass over a prolonged period (over weeks or months) (Table 3).

### 3.2. Chronic Animal Studies on Plant- versus Animal-Based Proteins Enrolling Young Individuals

Wróblewska et al. [31] demonstrated that young rats given soy proteins for 28 days had a lower lean mass gain than those fed whey proteins. At the muscular level, Combe et al. [23] and Pirman et al. [24] revealed that gastrocnemius and soleus muscle weights in young rats were significantly lower following 16 to 20 days of ad libitum consumption of cooked beans or cooked lentils compared to casein. This could be explained by the significantly lower muscle protein synthesis rate observed in rats fed legumes compared to casein. Combe et al. [23] and Pirman et al. [24] also evaluated intestinal protein synthesis rate after the ingestion of cooked legumes and showed that, compared to casein, the partitioning of dietary amino acid flux for protein synthesis went preferentially toward the intestinal tissues, to the detriment of liver and skeletal muscle tissues [23,24].

Taken together, these animal studies found that plant-based proteins have less of a capacity to improve lean and skeletal muscle mass than animal-based proteins [23,24,31]. Other studies have also evaluated the effect of the long-term consumption of plant- versus animal-based proteins on lean or skeletal muscle mass and muscle thickness in human subjects [28,37,38,39,98].

### 3.3. Chronic Clinical Studies on Plant- versus Animal-Based Proteins Enrolling Young Subjects

Hartman et al. [28] assessed the impact of soy intake with resistance exercise on lean mass accretion in young men and showed that the consumption of a drink containing ≈17.5 g soy protein during a 12 week period of resistance exercise training resulted in a 28% lower gain in lean body mass than when exercise was followed by an isonitrogenous milk protein drink [28]. Volek et al. [37] also demonstrated that the lean body mass gain in young men was 45% lower after consumption of 20 g of soy protein isolate compared to whey protein concentrate during a 36 week period of resistance exercise training. In contrast, Banaszek et al. [39] did not find any difference in body composition, especially lean mass, in adult subjects consuming either whey proteins or pea proteins during an 8 week period of high-intensity functional training. Note that Banaszek et al. [39] evaluated pea and whey protein supplements with overlapping leucine contents (whey protein: 2.2 g/dose, pea protein: 2.1 g/dose), which may explain their similar impact on lean mass changes after training [39].

All the animal and clinical studies discussed above evaluated the anabolic properties of single plant protein sources or commercial plant proteins (i.e., protein isolates [23,24,28,31,37,39,40,42,44,75]). Nevertheless, plant-based protein sources are rarely eaten ‘pure’ but are generally consumed as part of a meal containing various other sources of proteins.

### 3.4. Chronic Clinical Studies on Plant- versus Animal-Based Diets Enrolling Young and Older Subjects

Several papers have assessed the change in lean mass or muscle mass gain after the long-term consumption of plant-based meals (Table 3). A few chronic-intake studies have been conducted in older subjects to assess the potential role of plant-based protein diets in the prevention of sarcopenia [90,91,92,93,94,95,97]. Campbell et al. [90] assessed whether the consumption of an omnivorous (meat-containing) diet, during a 12 week period of resistance training, influenced changes in whole-body composition and skeletal muscle size in older men, compared to a lacto-ovo-vegetarian (meat-free) diet. Consumption of an omnivorous diet that provided 1.0 g protein/kg/d contributed to a greater gain in lean mass and skeletal muscle mass with resistance training in older men than a lacto-ovo-vegetarian diet that provided 0.78 g protein/kg/d. When daily dietary plant-based protein intake was 1.1 g protein/kg/d, i.e., higher than that assessed by Campbell et al. [90] for vegetarian diets (0.78 g/kg/d), the difference in muscle mass gains between the vegetarian and omnivorous diets was significantly reduced in healthy older men [91]. Studies have found that a plant-based protein diet could be an efficient strategy to enhance body lean mass, especially muscle mass, during a prolonged resistance exercise training when the amount of plant-based proteins consumed is 30 g/meal or greater [53,99,100]. Thus, plant-based proteins should be provided at sufficient amounts in each meal (i.e., >30 g/meal) to increase the potential to mitigate sarcopenia, as elderly subjects require a higher protein intake than young subjects [18]. Note that increasing the intake of a sole source of plant proteins deficient in certain essential amino acids could induce an increased rate of amino acid loss, i.e., increased deamination and oxidation [42]. This makes it important to blend different sources of plant proteins with complementary essential amino acid compositions in order to optimize plant-protein intake for older people. Isanejad et al. [93] conducted a cross-sectional and prospective cohort study that included 554 older women (65–72 years) and evaluated the association between different quantities and qualities of food protein and lean mass [93]. The results showed that a higher total protein intake (1.18 g/kg body weight/d) was positively associated with changes in lean mass and appendicular lean mass over 3 years of follow-up and showed a lower decrease in these parameters compared to a lower protein intake (0.79 g/kg body weight/d). The same observation was noted with animal protein intake, i.e., eggs, dairy products, poultry, and meat intakes [93]. Isanejad et al. [93] also showed that a higher intake of plant proteins (i.e., cereals, vegetables and fruits) was also significantly associated with a lower decrease in appendicular lean mass over 3 years of follow-up in the total elderly population [93], which is consistent with the results found by Chan et al. [94] and Huang et al. [95]. Sahni et al. [88] confirmed the results of Isanejad et al. [93] and showed that the total and animal protein intake was associated with an increase in lean leg mass. Nevertheless, over a 4 year period, Chan et al. [94] did not observe any association between total and animal protein intake and subsequent change in muscle mass in Chinese people aged 65 and older. Note that the mean relative total protein intake noted in this later study was 1.3 g/kg body weight [94], which is close to (and even higher than) the protein requirement recently proposed for older people >65 years (i.e., 1–1.2 g/kg body weight), to maintain muscle mass [101,102]. This high protein intake could explain, at least in part, the lack of association between protein intake and change in muscle mass [94]. Likewise, Verreijen et al. [97] did not find any association between the total, animal, or plant-protein intake with a 5 year change in the mid-thigh muscle cross-sectional area in older adults and suggested that using a single food-frequency questionnaire over two years might reduce the ability to detect this association in older adults due to the possibility that eating habits and protein intake may vary over this period [97].

Despite some contradictions, taken together, most of these studies suggest that the difference between the anabolic effects of plant- and animal-based proteins could be reduced with an adequate (i.e., increased) protein intake [91,93,95]. Therefore, an increase in plant protein intake could improve the ability of plant-based proteins to induce skeletal muscle mass gain and enhance their potential to support muscle mass maintenance in aging populations. Nevertheless, at similar protein intakes, most studies have reported a lower ability of plant-based protein sources to stimulate protein synthesis at the skeletal muscle level and induce muscle mass gain compared to animal-based protein sources, especially in older people [28,37,40,42,44,75]. The lower anabolic effect of plant-based protein sources is partly due to their lower digestibility [103] and their lower essential amino acid content, especially leucine [51], compared to animal proteins.

## 4. Main Strategies to Improve the Anabolic Properties of Plant-Based Protein Sources

### 4.1. Increased Protein Intake

As discussed above, consuming larger quantities of plant-based proteins per meal is expected to efficiently overcome their lower anabolic capacity and close the gap to the anabolic response observed with animal-based proteins. In this regard, Norton et al. [104] reported that the postprandial muscle protein synthesis rate after the ingestion of wheat proteins increased to the same extent as after a lower dose of whey proteins in young rats when the wheat protein dose was increased three-fold. In another study carried out in older subjects, Yang et al. [42] demonstrated that even the intake of a large amount of plant-based proteins (i.e., 40 g versus 20 g of soy proteins) was not as effective as whey proteins in improving postprandial muscle protein synthesis rates. Furthermore, they found a greater rate of amino acid oxidation following the ingestion of 40 g soy protein compared to ingestion of the same quantity of whey protein in older people. Therefore, when compared with animal-based proteins, amino acids derived from plant-based proteins were directed more toward oxidation than used for de novo muscle protein synthesis [42]. A more recent study showed that ingestion of a high dose of plant-based protein, i.e., 60 g of wheat protein, with an equivalent leucine content corresponding to 35 g of whey protein, induced a significant stimulation of postprandial muscle protein synthesis rates above the basal values in older men [44]. The increase in muscle protein synthesis was similar to animal-based protein and more efficient than a lower amount of wheat protein (i.e., 35 g). Increasing protein intake could help reach the essential amino acid requirements recommended for human diets. The appearance of amino acids into the circulation was more sustained after the ingestion of 60 g of wheat protein than 35 g of wheat protein, which induced a greater stimulation of postprandial muscle protein synthesis rates [44]. Note that aging is associated with blunted appetite, so the consumption of high quantities of plant proteins in order to stimulate muscle protein synthesis may not be a valid strategy for older individuals.

### 4.2. Supplementation with Limiting Amino Acids or Branched-Chain Amino Acids

Several studies have shown that leucine is able to stimulate specific intracellular signaling pathways related to mRNA translation and thereby acts as a signal nutrient in the stimulation of protein synthesis [12,76]. An animal study showed that supplementing wheat proteins with free leucine to match the leucine content present in whey proteins induced a similar postprandial muscle protein synthesis rate between the two proteins in young rats [29]. Likewise, a clinical study carried out by Engelen et al. [105] showed that fortifying soy proteins with branched-chain amino acids (leucine, isoleucine, and valine) also increased whole-body protein synthesis in favor of the peripheral body compartment, i.e., skeletal muscle, and decreased the splanchnic extraction and urea synthesis in healthy elderly. To our knowledge, the impact of fortifying plant-based diets with leucine on muscle mass in older subjects has not yet been studied. However, based on the relatively low leucine content in plant proteins compared to animal proteins, it is reasonable to posit that enriching plant-based proteins with free leucine would be an efficient strategy to enhance postprandial muscle protein synthesis response in elderly people.

A few rare studies have examined, in human populations, the effect of cereal fortification with lysine on the growth of children in developing countries. Zhao et al. [106] showed a significantly greater gain in the height and weight of children receiving lysine-fortified wheat flour, as well as significant improvements in some indicators of nutritional status and immune function [106]. Hussain et al. [107] also showed that fortifying wheat flour with lysine significantly improved the height and weight of children in the Pakistan population consuming a wheat-based diet (where more than 50% of the protein and calorie intake came from wheat). There have also been rare attempts to investigate the effect of supplementing legumes with sulfur amino acids on skeletal muscle mass gain [27]. Alonso et al. [27] demonstrated that supplementing extruded pea seeds with sulfur amino acids up to requirements for growth induced the same high levels of muscle protein synthesis and accretion as an isoproteic milk-based diet in young rats. Taken together, these studies showed that fortifying plant-based proteins with free amino acids (leucine, lysine, and sulfur amino acid) could be an efficient strategy to improve their anabolic effect. Nevertheless, further research is needed to assess the effect of amino acid-fortified plant-based proteins on postprandial muscle protein synthesis response in young and/or old subjects.

### 4.3. Protein Blending

#### 4.3.1. Blending Different Plant-Based Protein Sources

Cereal proteins (which are deficient in lysine) and legumes (which are deficient in sulfur amino acids) have complementary amino acid profiles [108]. In theory, mixing different plant-based proteins could compensate for the lower anabolic capacity of these protein sources [61], which means that combining various plant-based protein sources like cereals and legumes in the same food could improve essential amino acids composition to help meet the body’s needs [108] and even prove more efficient than fortification with free limiting amino acids. The free essential amino acids used to fortify plant-based proteins could be digested and absorbed faster than their constitutive amino acids, as suggested by Dardevet et al. [6] who produced an in-depth review of this postprandial desynchronization effect.

Very few studies have evaluated the effect of combining cereals with legumes on protein digestibility [109,110,111,112] and its impact on body protein metabolism [34,35,113]. Torres et al. [111,112] showed in young rats that the protein digestibility of wheat pasta enriched with 10% of fermented or germinated legume flour was significantly higher than that of conventional pasta (100% wheat semolina) but lower than that of casein. Acevedo-Pacheco and Serna-Saldívar [114] produced corn and wheat tortilla food matrices using 6% soy flour with a 35% higher lysine content than non-enriched products. As a result, protein retention was higher in rats consuming these legume-enriched tortillas than in rats consuming non-fortified products, but still lower than with casein [114]. Despite the increase in lysine content, the tortillas remained deficient in lysine (chemical score ≈ 55–64%), unlike casein, which had a balanced essential amino acid profile compared to the WHO/FAO/UNU recommended requirements [114,115]. Similarly, Laleg et al. [34] observed lower protein retention in young rats when fed diets containing wheat pasta enriched with 35% of faba bean flour that was still lysine-deficient (chemical score = 86%) compared to casein.

Taken together, these studies highlight the importance of fortifying cereal foods with legumes to improve their essential amino acid compositions and thus promote better body protein retention. Nevertheless, a further increase in the legume enrichment level in these products, to meet essential amino acid requirements according to body needs, could potentially achieve comparable protein retention to that induced by animal proteins like casein. However, to our knowledge, the nutritional benefits of this kind of combination have never been investigated in older subjects. Further research is warranted to test whether the strategy of blending legumes and cereals in the same foodstuff can efficiently limit muscle loss during aging.

#### 4.3.2. Blending Plant- and Animal-Based Protein Sources

Several studies have evaluated the effect of using a blend of 50% casein, 25% whey protein, and 25% soy protein on muscle protein synthesis in both young and older subjects [116,117,118,119]. Reidy et al. [118] showed in young subjects that after resistance exercise, muscle protein synthesis rates were not different between the group consuming 19 g of a mixture of plant and animal proteins (milk and soy) and the group consuming 18 g of whey protein. The activation of the signaling pathways driving the protein translation rate were also similar between the two groups [118]. These data are consistent with a more recent study carried out in older subjects (55–75 years old) [119], which showed that after resistance exercise, eating 30 g of a mixture of soy and milk proteins made it possible to achieve a similar aminoacidemia to that obtained after eating whey protein alone. Furthermore, muscle protein synthesis and degradation, and the net protein balance, were not different between the two groups [119]. Regardless of age, these studies show that combining plant with animal proteins can activate muscle protein anabolism in a similar way to high-nutritional-quality proteins, such as milk proteins (whey). We recently led an animal study that highlighted the important value of adding leucine-rich and fast-digestive protein (i.e., whey protein) to fermented dairy gels enriched with faba bean proteins [120]. In this work, body protein retention was 7% higher in young rats fed fermented faba bean–dairy gels containing whey proteins than in fermented faba bean gels made without whey proteins [120]. Furthermore, we observed higher whole-body protein retention in young rats fed the dairy gels enriched with 50% faba bean proteins than the retention observed by El-Moghazy et al. [121] in rats fed fermented faba bean proteins alone. Further research is needed to test the ability of this faba bean–dairy mixed gel to stimulate postprandial muscle protein synthesis and thereby support muscle mass in older subjects.

### 4.4. Using Plant Selections with More Balanced Amino Acid Composition

Using conventional plant breeding or genetic engineering to improve the nutritional quality of food crops (notably, to enhance their essential amino acid profile) may be an effective strategy to improve muscle protein synthesis response to the intake of plant-based proteins [53]. Quality protein maize (QPM) is not a genetically-modified maize. Its improved nutritional quality is produced by selectively breeding maize with a mutation of a gene named opaque-2, which leads to increased lysine and tryptophan contents [122,123]. QPM has a nearly two-fold higher lysine content compared to conventional maize [124], which at ≈ 42.5 mg/g protein is close to the WHO/FAO/UNU-recommended lysine requirements for adult humans (45 mg/g protein) [62,124]. QPM thus has a higher chemical score than conventional maize (94% versus 62%, respectively) [124]; (Table 2). To our knowledge, postprandial muscle protein synthesis after the consumption of QPM has not yet been assessed. However, the effectiveness of QPM in improving nutritional status has been evaluated in young children [125,126]. Compared to conventional maize, the growth rate was increased by 15% in the group of children that consumed QPM for one year [125]. The mid-upper-arm circumference of the young children significantly decreased from the basal value after the conventional maize diet, but this decrease was marginally less in the QPM group [125]. Therefore, using maize selections with a more balanced amino acid composition could be an efficient strategy to moderate undernutrition in populations in which this plant source is a significant part of the diet. More research is needed to evaluate the impact of eating plant selections with improved amino acid composition to increase postprandial muscle protein synthesis and enhance muscle mass after a long-term period of consumption.

## 5. Conclusions

High-quality protein consumption optimizes protein metabolism at both the whole-body and skeletal-muscle level, especially in older people. Plant-based protein sources that are rich in fiber and micronutrients may be valuable [127], but they have lower anabolic potential than animal-based proteins. Strategies to improve these properties by increasing protein intake or preferentially improving protein quality (i.e., their amino acid composition) include selective breeding, fortifying plant-based proteins with specific essential amino acids, mixing several plant proteins, and mixing plant- with animal-based protein sources. These strategies have been studied in younger individuals but now need to be examined in pathophysiological settings requiring high-quality food proteins to mitigate muscle loss. In the years to come, one of the chief challenges facing nutritionists is to reduce human suffering from skeletal muscle loss due to age or chronic disease. Utilizing cereal and legume composite mixes in product development could help improve nutritional properties, in terms not only of amino acid composition but also of fatty acid composition, fiber and phytochemical content, and vitamin and mineral density [128]. Formulated value-added products utilizing a mix of protein sources can support a healthier life, notably by helping to prevent chronic disease in aging. The successful utilization of cereals and legumes with added nutritional properties in foodstuffs will almost certainly open up new development perspectives for food industries.

## Figures and Tables

**Table 1:** Protein quality assessment based on protein sources.

Protein Type	Protein Digestibility (%)	Biological Value (%)	Net Protein Utilization (%)	PDCAAS	DIAAS
Animal source					
Red meat ^1^		80	73	92	
Casein ^1,3,6^	99	77	76–82	100	
Whey ^1^		104	92	100	
Milk ^1,4,6^	96	91	82	100	114
Egg ^1,4,6^	98	100	94	100	113
Plant source					
Black bean ^1,6,8^	70			75	
Cooked black bean ^7,8^	83			65	59
Soy flour ^5,8^	80			93	89(SAA)
Soy protein isolate^1,6^	98	74	61	100	
Green lentil ^3,4^	84			63	65
Yellow split pea ^4,6^	88			64	73
Cooked pea ^7^	89			60	58
Pea protein concentrate ^7^	99			89	82
Chickpea ^3,^^4^	89			74	83
Peanuts ^1^				52	
Roasted peanuts ^7^	98			51	43
Peanut butter ^3,4^	98			45	46
Whole grains ^2^				45	
Wheat ^3,5,6^	91	56–68	53–65	51	45(Lys)
Wheat gluten ^1^		64	67	25	
White bread ^4,6^	93			28	29
White rice ^4,6^	93			56	57
Cooked rice ^7^	87			62	60

^1^ Hoffman and Falvo [52]; ^2^ van Vliet et al. [53]; ^3^ Sarwar et al. [54]; ^4^ Marinangeli and House [55]; ^5^ Mathai et al. [56]; ^6^ ANSES [57]; ^7^ Rutherfurd et al. [58]; ^8^ Sarwar [59]. Abbreviations: PDCAAS: protein digestibility-corrected amino acid score; DIAAS: digestible indispensable amino acid score; Lys: lysine; SAA: sulfur amino acids.

**Table 2:** Essential amino acid scores of animal- and plant-based protein sources; adapted from Laleg et al. [60] and Gorissen and Witard [61].

		Plant-Based Proteins					Animal-Based Proteins			
	Wheat	Maize	Soybean	Pea	Faba Bean	Lentil	Whey	Casein	Milk	Beef
		Essential amino acid scores (%) ^1^								
Histidine	140	187	173	167	231	176	127	180	180	240
Isoleucine	137	127	157	153	112	154	213	167	170	167
Leucine	115	219	136	125	121	132	168	151	161	144
Lysine	31	62	147	182	158	160	204	169	153	207
Methionine + Cysteine	120	127	91	73	79	91	130	125	134	157
Phenylalanine + Tyrosine	290	300	277	267	247	263	227	343	313	280
Threonine	109	161	174	191	156	165	291	187	174	209
Valine	108	128	126	131	95	135	162	162	159	133

^1^ Scores are calculated based on recommendations for a healthy human adult [62].

**Table 3:** Studies carried out in the last ten years, assessing the anabolic properties of plant-based protein sources.

Study Type	Study (Reference)	Design	Method	Outcomes
Acute	Kanda et al. [30] Animal study	Young male Sprague-Dawley rats: *n* = 237 Oral administration of 3.1 g protein/kg BW Milk protein CC Whey protein CC Caseinate Soy protein CC	Isotope tracer	Soy proteins had an inferior effect on muscle protein synthesis after exercise compared with dairy proteins
	Norton et al. [29] Animal study	Young rats: *n* = 52 (Exp 1) *n* = 18 (Exp 2) Exp 2: 4 g meals (16% protein) Whey protein isolate Wheat gluten Wheat gluten+ Leu	Isotope tracer	Exp 2: Fortifying wheat with leucine to match the leucine content of whey diet induced similar anabolic responses, i.e., similar muscle protein synthesis rates
	Tang et al. [75] Clinical study	18 M Young subjects: 19–27 years Whey hydrolysate: 21.4 g Casein micelle: 21.9 g Soy protein isolate: 22.2 g	Isotope tracer	Muscle protein synthesis rates were in this order at rest condition: whey ≈ soy > casein after resistance exercise: whey > soy > casein
	Yang et al. [42] Clinical study	30 M Older subjects: 66–76 years 20 or 40 g Whey protein isolate Soy protein isolate	Isotope tracer	Soy protein isolate had less ability to stimulate muscle protein synthesis, compared to whey protein isolate under both rested and post-exercise conditions
	Gorissen et al. [44] Clinical study	60 M Older subjects: 70–72 years 35 or 60 g Whey protein isolate Micellar casein Wheat protein hydrolysate	Isotope tracer	Muscle protein synthesis rates were lower after ingesting 35 g wheat protein than after the same amount of casein. Ingesting a larger quantity of wheat protein (i.e., 60 g) substantially improved muscle protein synthesis rates in elderly men
	Laleg et al. [34] Animal study	Young male Wistar Rats *n* = 50 *Ad libitum* consumption of isoproteic and isocaloric diets for 3 weeks Faba bean-enriched pasta Wheat gluten pasta Casein	Echo-MRI	Rats fed legume-enriched pasta or wheat gluten pasta had a lower LM than rats fed casein
Chronic	Volek et al. [37] Clinical study	63 M + F Young subjects: 18–35 years Daily consumption of supplements containing whey or soy proteins + Whole-body periodized resistance training program for 36 weeks	DXA	Daily supplementation with whey was more effective than isoproteic and isocaloric supplement containing soy protein in enhancing LM during resistance training
	Mobley et al. [38] Clinical study	75 F Young subjects: 20–22 years Daily consumption (twice) of supplements containing whey or soy proteins + Whole-body resistance training (3 d/week) for 12 weeks	DXA	Whey and soy supplement groups showed similar increases in total body skeletal muscle mass and type I and II fiber cross-sectional area during resistance training
	Banaszek et al. [39] Clinical study	15 M + F Young and adult subjects: 26–51 years Consumption of supplements containing whey or pea proteins on training day + High-intensity functional training (4 sessions/week) for 8 weeks	BIA	Ingestion of whey and pea protein produced similar outcomes in measurements of body composition, especially LM and muscle thickness
	Chan et al. [94] Clinical study	1411 M + 1315 F Older subjects: 65 years and older L (4 years), FFQ	DXA	Higher plant (but not total and animal) protein intakes were associated with reduced muscle loss
Chronic	Isanejad et al. [93] Clinical study	554 F Older subjects: 65–72 years L (3 years); 3 d food record	DXA	Higher total and animal protein intakes were associated with increased LM and ALM Higher plant protein intake was associated with less reduction in ALM
	Sahni et al. [88] Clinical study	1139 M + 1497 F Young and older subjects: 29–86 years CS; FFQ	DXA	Higher total and animal (but not plant) protein intakes were associated with higher LM in the leg
	Miki et al. [96] Clinical study	168 M + F with type 2 diabetes Older subjects: ≥ 65 years CS; BDHQ	BIA	Total and plant protein intakes were positively associated with skeletal muscle mass
	Huang et al. [95] Clinical study	327 M + F Older subjects: 66–76 years CS; FFQ	BIA	Low total and plant protein intakes were associated with a higher risk for low muscle mass
	Verreijen et al. [97] Clinical study	3075 M + F Older subjects: 70–79 years L (5 years); FFQ	DXA	Higher total, animal and plant protein intakes were not associated with changes in mid-thigh-muscle CSA
	Mangano et al. [89] Clinical study	2986 M + F Young and older subjects: 19–72 years L; FFQ Food clusters 1. Fast food 2. Red meat 3. Fish 4. Chicken 5. Low-fat milk 6. Legumes	DXA	Individuals in the legume protein food cluster had significantly lower ALM compared with subjects in all other protein food clusters. No associations between protein clusters and any musculoskeletal outcomes in adjusted models.

Abbreviations: M: male; F: female; CS: cross-sectional; L: longitudinal; FFQ: food-frequency questionnaire; BDHQ: brief-type self-administered diet history questionnaire; LM: lean mass; ALM: appendicular lean mass; CC: concentrate; BW: body weight; Exp: experiment; DXA: dual-energy X-ray absorptiometry; BIA: bioelectrical impedance analysis; CSA: cross-sectional area.
